# Distributed Joint Source-Channel Coding Using Quasi-Uniform Systematic Polar Codes

**DOI:** 10.3390/e20100806

**Published:** 2018-10-22

**Authors:** Liqiang Jin, Hongwen Yang

**Affiliations:** School of Information and Communication Engineering, Beijing University of Posts and Telecommunications, Beijing 100876, China

**Keywords:** correlated sources, noisy independent channels, distributed joint source-channel coding, quasi-uniform systematic polar codes

## Abstract

This paper proposes a distributed joint source-channel coding (DJSCC) scheme using polar-like codes. In the proposed scheme, each distributed source encodes source message with a quasi-uniform systematic polar code (QSPC) or a punctured QSPC, and only transmits parity bits over its independent channel. These systematic codes play the role of both source compression and error protection. For the infinite code-length, we show that the proposed scheme approaches the information-theoretical limit by the technique of joint source-channel polarization with side information. For the finite code-length, the simulation results verify that the proposed scheme outperforms the distributed separate source-channel coding (DSSCC) scheme using polar codes and the DJSCC scheme using classic systematic polar codes.

## 1. Introduction

Polar codes, invented by Arikan [1] using a technique called channel polarization, are capable of achieving the symmetric capacity of any binary-input discrete memoryless channel (B-DMC) with low encoding and decoding complexity. Afterwards, the concept of source polarization was introduced in [2] as the complement of channel polarization. One immediate application of source polarization is the design of polar codes for source coding. Since the polarization phenomenon exists on both source and channel sides, it is of natural interest to integrate channel polarization and source polarization for joint source-channel coding (JSCC). From the perspective of information theory, this interest is motivated by the fact that the error exponent of JSCC is better than that of separate source-channel coding (SSCC) [3], which may indicate a performance loss of SSCC under the finite code-length. From a practical point of view, two main shortcomings of SSCC resulting in an unsatisfactory performance are summarized as follows:

- With finite code-length, channel decoding error is unavoidable, such error may be disastrous for the source decoder.
- With finite code-length, in the output of source coding may exist residual redundancy, such redundancy is not exploited by the channel decoder to further improve the performance.

Therefore, the JSCC scheme, which takes into consideration both source redundancy and channel error jointly, may have certain advantages over SSCC.

In recent years, JSCC has attracted an increasing number of attention among researchers. The JSCC schemes based on turbo codes, LDPC codes, and polar codes can be found in [4,5,6,7], and [8,9,10], respectively. In particular, the authors of [11] proposed a class of systematic polar codes (SPCs), called quasi-uniform SPCs (QSPCs), and showed that QSPCs are optimal for the problem of JSCC with side information and outperform classic SPCs [12].

For the distributed source coding (DSC) problem, the Slepian-Wolf theorem [13] states that for two or more correlated sources, lossless compression rates of joint encoding can be achieved with separate encoding if a joint decoder is used at the receiver. This theorem has been known for a long time, but the practical DSC scheme was only recently proposed by Pradhan and Ramchandran using syndromes [14]. Inspired by the syndromes approach, a large number of DSC schemes have sprung up based on powerful channel codes (CCs) such as turbo codes, LDPC codes, polar codes [15,16,17]. However, to obtain a good performance, these syndromes-based schemes usually need a considerably long code-length ($10^{4}∼10^{5}$) and are sensitive to channel errors. Alternatively, the design of DSC scheme can be based on parity approach where a systematic code is used to encode the source and the source is recovered by parity bits. These kinds of schemes can be extended to distributed JSCC (DJSCC) [18,19]. Our work falls into this category. Specifically, we focus on the design of parity-based DJSCC using polar/polar-like codes, where only parity bits are transmitted over the noisy channels. Each distributed source has one encoder to encode source message for both compression and error protection.

The main contributions of this paper are summarized as follows. We propose a new class of polar-like codes, called punctured QSPCs, and show its asymptotic optimality. Then we propose a DJSCC scheme using two polar-like codes and show that the proposed scheme approaches achievable rates.

The rest of this paper is organized as follows. The system model is given in Section 2. Section 3 introduces QSPCs and punctured QSPCs. In Section 4, we propose a DJSCC scheme based on two polar-like codes and analyze its performance limit. Section 5 presents the simulation results to verify the advantage of the proposed scheme and in Section 6 we conclude this paper.

## 2. System Model

Consider the problem of transmitting distributed sources $V_{1},V_{2},\ldots ,V_{s}$ over independent symmetric B-DMCs $W_{1},W_{2},\ldots ,W_{s}$ to a common destination. The message from source $V_{i},i=1,2,\ldots ,s,$ is denoted by a binary vector $v_{i}=\left[v_{i}^{1},v_{i}^{2},\ldots ,v_{i}^{K}\right]$ of length *K*. We assume that $v^{j}=\left[v_{1}^{j},v_{2}^{j},\ldots ,v_{s}^{j}\right]$ for different j∈{1,2,…,K} are i.i.d. (identically and independently distributed) vectors drawn from ${\left\{0,1\right\}}^{s}$ with a common probability distribution $P_{V_{1},V_{2},\ldots V_{s}}$.

From the Slepian-Wolf theorem, the compression rate $R=(R_{1},R_{2},\ldots ,R_{s})$ is achievable if

(1) $$\sum_{i\in T}R_{i}\geq H\left(v_{T}^{j}|v_{T^{c}}^{j}\right)$$

for any T⊆{1,2,…,s}, where H(·) is entropy function, $T^{c}=\left\{1,2,\ldots ,s\right\}\setminus T$ is the complementary set of T, $v_{T}^{j}$ and $v_{T^{c}}^{j}$ denote, respectively, the sub-vectors of $v^{j}$ indexed by T and $T^{c}$. This achievable compression region is also known as the Slepian-Wolf region $R_{SW}$.

Let ${\tilde{R}}_{i}={\tilde{N}}_{i}/K$ be the rate, measured in the number of channel-use per source-symbol, where ${\tilde{N}}_{i}$ is the number of channel uses of source $V_{i}$ for transmitting $v_{i}$. Let $C_{i}$ denote the channel capacity of $W_{i}$. Since the source-channel separation theorem still holds for this problem [20], reliable transmission is possible if $\tilde{R}=\left({\tilde{R}}_{1},{\tilde{R}}_{2},\ldots ,{\tilde{R}}_{s}\right)$ falls into the achievable rate region $R_{\mathrm{rate}}$, which is defined as

(2) $$R_{\mathrm{rate}}=\left\{\tilde{R}|{\tilde{R}}_{i}\geq 0,R_{\mathrm{SW}}\cap R_{c}\left(\tilde{R}\right)\neq \emptyset \right\},$$

where $R_{c}\left(\tilde{R}\right)$ is the set of all points $\left(R_{1},R_{2},\ldots ,R_{s}\right)$ satisfying

(3) $$0\leq R_{i}\leq {\tilde{R}}_{i}C_{i}$$

for i=1,2,…,s.

Given ${\tilde{R}}_{1},{\tilde{R}}_{2}\in R_{\mathrm{rate}}$ and 0≤λ≤1, by the definition of convex sets, we can readily prove that $\lambda {\tilde{R}}_{1}+\left(1-\lambda \right){\tilde{R}}_{2}\in R_{\mathrm{rate}}$ as follows. There obviously exist $R_{1}^{*}\in R_{\mathrm{SW}}\cap R_{c}\left({\tilde{R}}_{1}\right)$ and $R_{2}^{*}\in R_{\mathrm{SW}}\cap R_{c}\left({\tilde{R}}_{2}\right)$. Due to the convexity of $R_{\mathrm{SW}}$, $\lambda R_{1}^{*}+\left(1-\lambda \right)R_{2}^{*}\in R_{\mathrm{SW}}$. Besides, it can be observed that

(4) $$\lambda R_{1}^{*}+\left(1-\lambda \right)R_{2}^{*}\in R_{c}\left(\lambda {\tilde{R}}_{1}+\left(1-\lambda \right){\tilde{R}}_{2}\right).$$

This indicates that a point at least exists in $R_{\mathrm{SW}}\cap R_{c}\left(\lambda {\tilde{R}}_{1}+\left(1-\lambda \right){\tilde{R}}_{2}\right)$, and $R_{\mathrm{SW}}\cap R_{c}\left(\lambda {\tilde{R}}_{1}+\left(1-\lambda \right){\tilde{R}}_{2}\right)\neq \emptyset$. Therefore, $\lambda {\tilde{R}}_{1}+\left(1-\lambda \right){\tilde{R}}_{2}\in R_{\mathrm{rate}}$ and the achievable rate region of $R_{\mathrm{rate}}$ is convex.

**Figure 1:**
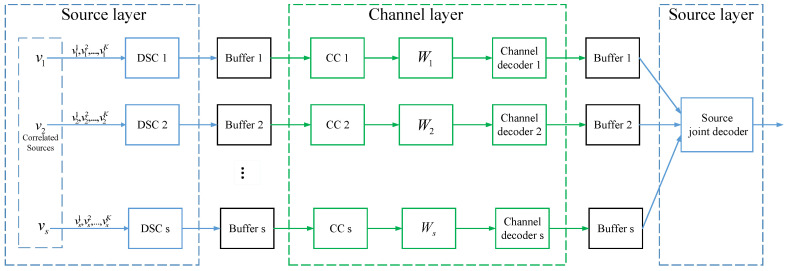
Distributed separate source-channel coding (DSSCC) scheme.

**Figure 2:**
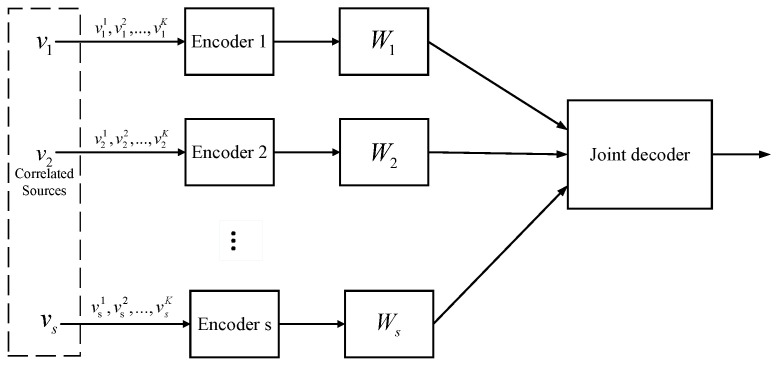
Distributed joint source-channel coding (DJSCC) scheme.

To solve this transmission problem, one option is distributed SSCC (DSSCC) scheme as shown in Figure 1. The message from each source is first compressed into compressed bits with DSC. Then each source encodes its compressed bits into channel code bits with CCs and transmit these channel code bits over its channel. At the destination, the received signals are first decoded by channel decoders, and then decoded bits are jointly decompressed by a source joint decoder. There is no interaction between the source layer and the channel layer in the sense of encoding and decoding. Such a DSSCC scheme is asymptotically optimal when the code-length goes to infinity. However, with finite code-length, such source-channel separate schemes are generally sensitive to the residual errors of channel decoder, and thus are ineffective in some scenarios such as wireless sensor networks (WSNs). To address this problem, in this paper we propose a DJSCC scheme using polar-like codes to achieve the corner point in $R_{\mathrm{rate}}$, i.e.,

(5) $${\tilde{R}}_{i}=\frac{H(v_{i}^{j}|v_{1}^{j}v_{2}^{j}\ldots v_{i-1}^{j})}{C_{i}}$$

for i=1,2,…,s. As shown in Figure 2, each source is equipped with only one encoder to encode the source message for both compression and error protection.

Note that the order of the chain rule $v_{1}^{j}v_{2}^{j}\ldots v_{s}^{j}$ in (5) has no effect on the code construction and overall performance, hence the ascending order is considered in this paper. It is also obvious that multiple points in $R_{\mathrm{rate}}$ can be achieved with time-sharing operations, provided that DJSCC schemes for corner points have been well constructed.

## 3. QSPC and Punctured QSPC

In this section, we briefly review QSPC and propose punctured QSPC. At first, two index sets and their properties are presented. Consider a binary sequence L of length $N=2^{n}$. The coordinates of the ones in L are quasi-uniformly distributed and denoted by the index set Q(N,K) or $\bar{Q}\left(N,K\right)$, which is defined as

(6) $$\begin{array}{c} Q\left(N,K\right)=\{b=1+\sum_{i=0}^{n-1}b_{i}2^{i}|1+\sum_{i=0}^{n-1}b_{i}2^{n-1-i}\in \left\{i:N-K+1\leq i\leq N\right\},b_{i}\in \left\{0,1\right\}\},\\ \bar{Q}\left(N,K\right)=\left\{b=1+\sum_{i=0}^{n-1}b_{i}2^{i}|1+\sum_{i=0}^{n-1}b_{i}2^{n-1-i}\in \left\{i:1\leq i\leq K\right\},b_{i}\in \left\{0,1\right\}\right\}. \end{array}$$

The property of this sequence is given as Proposition 1.

**Proposition** **1.** *The coordinates of the ones in $L_{M}={\underset{︸}{LL\ldots L}}_{M}\;\left(M=2^{m}\right)$ are Q(MN,MK) and $\bar{Q}\left(MN,MK\right)$ if the coordinates of the ones in L are Q(N,K) and $\bar{Q}\left(N,K\right)$, respectively.*

**Figure 3:**
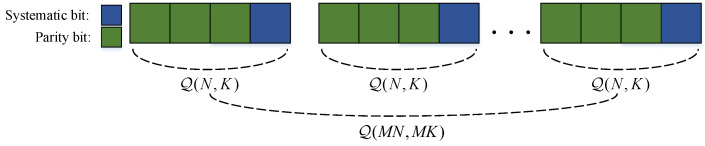
The codeword structure of quasi-uniform systematic polar code (QSPCs) with N=4,K=1.

**Figure 4:**
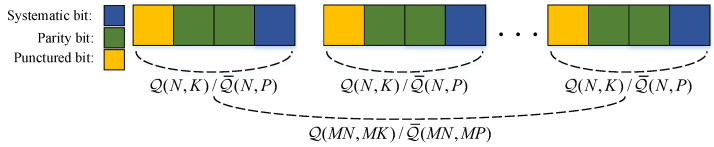
The codeword structure of punctured QSPCs with N=4,K=1,P=1.

An example for index sets is illustrated in Figure 3 and Figure 4. The coordinates of the ones in L=0001 and $\bar{L}=1000$ are Q(4,1)={4} and $\bar{Q}\left(4,1\right)=\left\{1\right\}$, respectively. For $L_{2}=00010001$ and ${\bar{L}}_{2}=10001000$, the coordinates of ones are Q(8,2)={4,8} and $\bar{Q}\left(8,2\right)=\left\{1,5\right\}$, respectively.

### 3.1. QSPC

A class of SPCs, called QSPCs, were introduced for the problem of JSCC with side information. For a (MN,MK) QSPC ($M=2^{m},N=2^{n},K\leq N$), systematic bits $x_{B}$ are first encoded by

(7) $$\begin{array}{cc} {\tilde{u}}_{\tilde{A}} & =\left(x_{B}-u_{{\tilde{A}}^{c}}{\tilde{G}}_{MN}\left({\tilde{A}}^{c}B\right)\right){\left({\tilde{G}}_{MN}\left(\tilde{A}B\right)\right)}^{-1}\\ & =x_{B}{\left({\tilde{G}}_{MN}\left(\tilde{A}B\right)\right)}^{-1}, \end{array}$$

where $\tilde{A}=\left\{i|MN-MK-1\leq i\leq MN\right\},B=Q\left(MN,MK\right)$, and ${\tilde{G}}_{MN}\left(\tilde{A}B\right)$ denotes the sub-matrix of ${\tilde{G}}_{MN}$ with the rows indexed by $\tilde{A}$ and the columns indexed by B. Then the codeword is obtained by

(8) $$x=\left({\tilde{u}}_{\tilde{A}}{\tilde{G}}_{MN}\left(\tilde{A}\right)\right)⊕\left({\tilde{u}}_{{\tilde{A}}^{c}}{\tilde{G}}_{MN}\left({\tilde{A}}^{c}\right)\right),$$

where ${\tilde{G}}_{MN}\left(\tilde{A}\right)$ and ${\tilde{G}}_{MN}\left({\tilde{A}}^{c}\right)$ denote the rows of ${\tilde{G}}_{MN}$ indexed by $\tilde{A}$ and ${\tilde{A}}^{c}$, respectively. The generator matrix is given by

(9) $${\tilde{G}}_{MN}=DG_{MN}=DB_{MN}{\left[\begin{array}{cc} 1 & 0\\ 1 & 1 \end{array}\right]}^{⊗(m+n)},$$

where $B_{MN}$ is a bit-reversal matrix, and *D* is an invertible bit-swap coding matrix (refer to Section IV.A of [11] for details). It can be seen that $x=\tilde{u}{\tilde{G}}_{MN}=uG_{MN}$ and $u=\tilde{u}D$.

For this code, the typical decoder of polar codes attempts to decode u, then the codeword x is obtained by $x=uG_{MN}$ or $x=uD^{-1}{\tilde{G}}_{MN}$. The entropies/BERs (bit error rate) of u are determined by underlying channels and the sources. With the aid of density evolution, we can locate those bits $u_{A}$ which have low entropies/BERs. However, the reversibility of $G_{AB}$ cannot be guaranteed, i.e., it is impossible to construct QSPCs via original polar coding. The bit-swap coding *D* is then proposed to change the information bits from $u_{A}$ to ${\tilde{u}}_{\tilde{A}}$ and modify the generator matrix ${\tilde{G}}_{MN}=DG_{MN}$. This operation ensures that ${\tilde{G}}_{\tilde{A}B}$ is always invertible. The block error rate (BLER) of x under the SC decoder can be calculated by

(10) $$P_{\mathrm{BLER}}=1-\prod_{i\in A}\left[1-P_{e}\left(u_{i}\right)\right],$$

where $P_{e}\left(u_{i}\right)$ is the BER of $u_{i}$.

Consider a source message $v=[v^{1},v^{2},\ldots ,v^{MK}]$ with side information $w=[w^{1},w^{2},\ldots ,w^{MK}]$. When v is encoded by a (MN,MK) QSPC for JSCC, only parity bits $x_{B^{c}}$ are transmitted over the noisy channel W(y|x). Due to B=Q(MN,MK), the codeword structure can be depicted in Figure 3, and Theorem 1 can be obtained as follows.

**Theorem** **1 ([11]).** *For any $0\leq \epsilon <\frac{1}{2}$,*

(11) $$\begin{array}{l} \lim_{M\to \infty }\frac{1}{MN}\left|\{i:H\left(u_{i}|yu_{1:i-1}\right)\in \left[0,\epsilon \right)\}\right|=\lim_{M\to \infty }\frac{1}{MN}\left|\left\{i:Z\left(u_{i}|yu_{1:i-1}\right)\in \left[0,\epsilon \right)\right\}\right|\\ =1-\frac{KH(v|w)+(N-K)H(x|y)}{N},\\ \lim_{M\to \infty }\frac{1}{MN}|\left\{i:H\left(u_{i}|yu_{1:i-1}\right)\in \left(1-\epsilon ,1\right]\right\}|=\lim_{M\to \infty }\frac{1}{MN}\left|\left\{i:Z\left(u_{i}|yu_{1:i-1}\right)\in \left(1-\epsilon ,1\right]\right\}\right|\\ =\frac{KH(v|w)+(N-K)H(x|y)}{N}, \end{array}$$

*where |·| denotes the cardinality of set, Z(·|·) is the source Bhattacharyya parameter [2], $y_{B}=w$ and $y_{B^{c}}$ are channel outputs of W(y|x), and 1:i−1 denotes the set of {1,2,…,i−1}.*

Note that the theorem shows that $P_{\mathrm{BLER}}$ tends to 0 as *M* goes to infinity, if H(v|w)/(1−H(x|y))=H(v|w)/C≤(N−K)/K, because $Z(u_{i}|yu_{1:i-1})$ is an upper-bound of $P_{e}\left(u_{i}\right)$. Further, if the supermartingale is established for $Z(u_{i}|yu_{1:i-1})$, we have $P_{\mathrm{BLER}}\leq 2^{-M^{\beta }}\to 0$, for any $\beta \in (0,\frac{1}{2})$ [21].

### 3.2. Punctured QSPC

By puncturing MP parity bits from a (MN,MK) QSPC code, we can construct a punctured QSPC code with a lower rate ${\tilde{R}}_{i}$ (in channel-use per source-symbol). This punctured QSPC is dominated by the parameter (MN,MK,MP). As depicted in Figure 4, there are three kinds of bits (systematic bits, parity bits, and punctured bits) in the codeword, and the coordinates of punctured bits are $\bar{Q}\left(MN,MP\right)$. At the stage of code construction, punctured bits are regarded as the inputs of the zero-capacity channel to calculate BERs of u and bit-swap coding. When this punctured QSPC is used to encode source message $v=[v^{1},v^{2},\ldots ,v^{MK}]$ with side information $w=[w^{1},w^{2},\ldots ,w^{MK}]$, Theorem 2 is obtained similarly as follows.

**Theorem** **2.** *For any $0\leq \epsilon <\frac{1}{2}$,*

(12) $$\begin{array}{l} \lim_{M\to \infty }\frac{1}{MN}|\left\{i:H\left(u_{i}|yu_{1:i-1}\right)\in \left[0,\epsilon \right)\right\}|=\lim_{M\to \infty }\frac{1}{MN}\left|\left\{i:Z\left(u_{i}|yu_{1:i-1}\right)\in \left[0,\epsilon \right)\right\}\right|\\ =1-\frac{KH(v|w)+(N-K-P)H(x|y)+P}{N},\\ \lim_{M\to \infty }\frac{1}{MN}|\left\{i:H\left(u_{i}|yu_{1:i-1}\right)\in \left(1-\epsilon ,1\right]\right\}|=\lim_{M\to \infty }\frac{1}{MN}\left|\left\{i:Z\left(u_{i}|yu_{1:i-1}\right)\in \left(1-\epsilon ,1\right]\right\}\right|\\ =\frac{KH(v|w)+(N-K-P)H(x|y)+P}{N}, \end{array}$$

*where $y_{\bar{Q}\left(MN,MP\right)}=\emptyset$ because of puncturing.*

**Proof** **of Theorem 2.** The codeword structure of Figure 4 ensures that $Z(u_{i}|yu_{1:i-1})$ and $H(u_{i}|yu_{1:i-1})$ converge to either 0 or 1, as *M* goes to infinity, which is similar to QSPC. The total entropy can be calculated by

(13) $$\begin{array}{c} \sum_{i=1}^{MN}H\left(u_{i}|yu_{1:i-1}\right)=\sum_{i=1}^{MN}H\left(x_{i}|y_{i}\right)=M\left(N-K-P\right)H\left(x|y\right)+MKH\left(v|w\right)+MP. \end{array}$$

Therefore, the fraction of the high entropy part (H≈1) is

(14) $$\frac{1}{MN}\left(M(N-P-K)H(x|y)+MKH(v|w)+MP\right)=\frac{1}{N}\left(N-P-K\right)H\left(x|y\right)+\frac{1}{N}KH\left(v|w\right)+\frac{P}{N},$$

and the fraction of the low entropy part (H≈0) is $1-\frac{1}{N}\left(N-P-K\right)H\left(x|y\right)-\frac{1}{N}KH\left(v|w\right)-\frac{P}{N}$. □

This theorem also shows that $P_{\mathrm{BLER}}$ of punctured QSPC tends to 0 as *M* goes to infinity, if H(v|w)/(1−H(x|y))=H(v|w)/C≤(N−K−P)/K. Since punctured bits contributes zero channel-use, punctured QSPC has no performance loss. After the establishment of supermartingale for $Z(u_{i}|yu_{1:i-1})$, we also have $P_{\mathrm{BLER}}\leq 2^{-M^{\beta }}\to 0$, for any $\beta \in (0,\frac{1}{2})$.

## 4. Proposed DJSCC Scheme and Performance Limit

In this section, we propose a DJSCC scheme based on the previous construction of two polar-like codes, and prove its asymptotic optimality.

### 4.1. Proposed DJSCC Scheme

To achieve the corner point (5), we turn this problem into the problem of JSCC with side information at the receiver, and solve it with QSPC and punctured QSPC. The proposed DJSCC scheme consists of *s* encoders and one joint decoder, as shown in Figure 2.

Define

(15) $${\tilde{R}}_{\max}=\max_{i\in \{1,2,\ldots ,s\}}{\tilde{R}}_{i}$$

and

(16) $$i_{\max}=\underset{i\in \{1,2,\ldots ,s\}}{\mathrm{argmax}}{\tilde{R}}_{i}.$$

**Figure 5:**
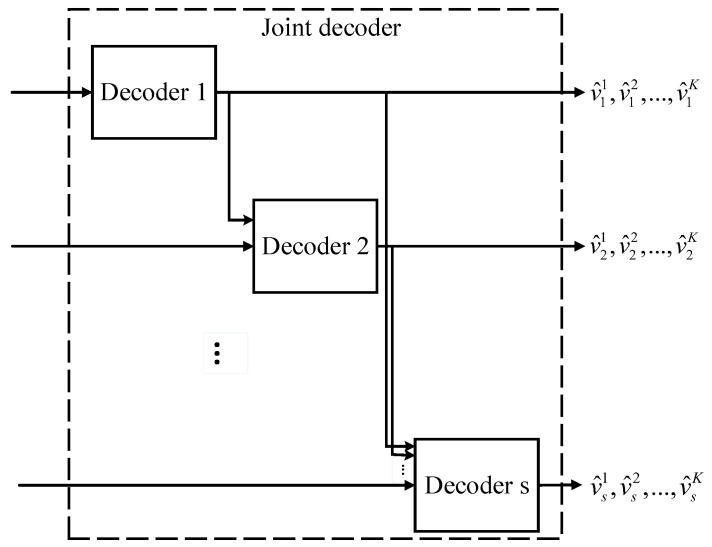
Joint decoder.

For source $V_{i}$, the previous sources $V_{1},V_{2},\ldots ,V_{i-1}$ are completely regarded as side information to construct QSPC and punctured QSPC. For the source $V_{i_{\max}}$, its message $v_{i_{\max}}$ is encoded by a (MN,MK) QSPC. For sources $V_{i},i\neq i_{\max}$, the message $v_{i}$ is encoded by a $\left(MN,MK,MP_{i}\right)$ punctured QSPC to adapt a lower rate ${\tilde{R}}_{i}$. At the destination, a joint decoder is used to recover the sources, as shown in Figure 5. The joint decoder consists of *s* conventional polar decoders working successively. The hard decisions ${\hat{v}}_{1},{\hat{v}}_{2},\ldots ,{\hat{v}}_{i-1}$ from decoder 1,2,…,i−1 are fed to *i*-th decoder for calculating log likelihood ratios (LLRs) of systematic bits. For parity bits, the LLRs are calculated with channel observations.

### 4.2. Performance Limit

Based on the analysis in Section 3, both QSPC and punctured QSPC can achieve the information-theoretical limit H/C in channel-use per source-symbol. Therefore, the proposed scheme is asymptotically optimal, as long as the rates can be arbitrarily approached. The following lemma shows that the QSPC and punctured QSPC can approach the rate limits.

**Lemma** **1.** *The (MN,MK) QSPC asymptotically approaches the rate ${\tilde{R}}_{i_{\max}}$ and the $\left(MN,MK,MP_{i}\right)$ punctured QSPC asymptotically approaches arbitrary rate ${\tilde{R}}_{i}\leq {\tilde{R}}_{i_{\max}}$.*

**Proof** **of Lemma 1.** Let *K* be the integer part of $N/({\tilde{R}}_{i}+1)$, then we have

(17) $$\begin{array}{c} N=K\left({\tilde{R}}_{i}+1\right)+\epsilon ,\;0\leq \epsilon <{\tilde{R}}_{i}+1 \end{array}$$

and

(18) $$\begin{array}{c} {\tilde{R}}_{\mathrm{peak}}=\frac{N-K}{K}=\frac{K({\tilde{R}}_{i}+1)+\epsilon -K}{K}={\tilde{R}}_{i}+\frac{\epsilon }{K}. \end{array}$$

Since $\lim_{K(N)\to \infty }{\tilde{R}}_{\mathrm{peak}}={\tilde{R}}_{i}+\lim_{K(N)\to \infty }\frac{\epsilon }{K}={\tilde{R}}_{i}$, the gap between ${\tilde{R}}_{\mathrm{peak}}$ and ${\tilde{R}}_{i}$ can be arbitrarily small when *N* is sufficiently large. Besides, the minimum step of adjustable rate by puncturing $\Delta =\frac{1}{K}$ is getting smaller with increasing *N*. This indicates that the punctured QSPC can also arbitrarily approach a rate less than ${\tilde{R}}_{\mathrm{peak}}$ when *N* is large enough. □

## 5. Simulation Results

In the previous section, we have proposed a DJSCC scheme and shown its asymptotic optimality. In this section, we present the simulation results to verify the performance of the proposed scheme under finite code-length.

In the simulation, two sources $V_{1},V_{2}$ are considered and $\mathrm{Pr}\{v_{2}^{j}|v_{1}^{j}\}$ is modeled as binary symmetric channel (BSC) with a crossover probability q=0.11, and $\mathrm{Pr}\{v_{1}^{j}=0\}=0.5$. The transmission channels $W_{1},W_{2}$ are BI-AWGN channels with the same signal-to-noise ratio (SNR). The (64·32,64·10) QSPC and the (64·32,64·10,64·11) punctured QSPC are constructed with density evolution. The QSPC and the punctured QSPC are respectively used to encode $v_{1}$ and $v_{2}$ for DJSCC. For this DJSCC scheme, the rates are $\tilde{R}=\left({\tilde{R}}_{1},{\tilde{R}}_{2}\right)=\left(2.2,1.1\right)$ in channel-use/source-symbol. The Shannon limits for both two sources are approximately -0.4 dB, which are respectively obtained by $MK\cdot H\left(v_{1}^{j}\right)\leq M\left(N-K\right)\cdot C\left(\mathrm{SNR}\right)$ and $MK\cdot H\left(v_{2}^{j}|v_{1}^{j}\right)\leq M\left(N-K-P\right)\cdot C\left(\mathrm{SNR}\right)$, where C(SNR) is the capacity of BI-AWGN channels.

**Figure 6:**
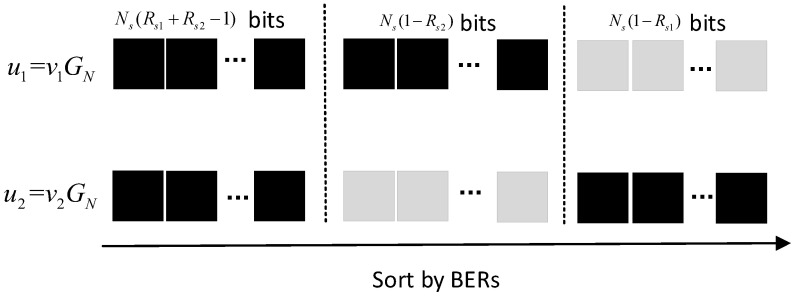
Bit selection of distributed source coding (DSC) based on polar codes.

As a reference, we consider a DSSCC scheme where both DSC and CC are designed using polar codes of length $N_{s}=N_{c}=1024$. The source compression rate (in bit/source-symbol) of DSC $R_{si}$, and the channel code rate (in bit/channel-use) of CC $R_{ci}$ for source $V_{i},i=1,2$ are illustrated in Table 1. The rate of this DSSCC scheme can be calculated by

(19) $${\tilde{R}}_{i}=\frac{R_{si}}{R_{ci}},\;i=1,2$$

in channel-use/source-symbol. In the source layer, each $N_{s}$ bits of source message are divided into a block for DSC and the polar code based DSC is designed as follows. After polar transform $u=\left[v_{1}^{1}⊕v_{2}^{1},v_{1}^{2}⊕v_{2}^{2},\ldots ,v_{1}^{N_{s}}⊕v_{2}^{N_{s}}\right]G_{N}$, the BERs $P_{e}\left(u_{i}\right)$ of u are calculated with density evolution. As shown in Figure 6, both $u_{1}=v_{1}G_{N}$ and $u_{2}=v_{2}G_{N}$ are sorted in a descend order according to $P_{e}\left(u_{i}\right)$, and the bits represented by black boxes are compressed bits that will be fed into the buffer for CC. At the receiver, the decoder firstly tries to recover $u_{1}⊕u_{2}$ with LLRs $\log\frac{1-q}{q}$, then the bits represented by gray boxes are reconstructed with some modulo-2 additions. Finally, the sources are obtained by ${\hat{v}}_{1}={\hat{u}}_{1}G_{N}$ and ${\hat{v}}_{2}={\hat{u}}_{2}G_{N}$. In the channel layer, each $N_{c}R_{ci}$ compressed bits in the buffer are divided into a block for $\left(N_{c},N_{c}R_{ci}\right)$ CC based on polar codes.

Besides, we consider the DJSCC based on classic SPCs as a reference. For source $V_{1}$, the (64·32,64·10) SPC is constructed via Gaussian approximation where the underlying channel is BI-AWGN channels with the equivalent capacity $C_{equ1}=\frac{K}{N}\cdot \left(1-H\left(v_{1}^{j}\right)\right)+\frac{N-K}{N}C_{1}=\frac{N-K}{N}C_{1}$. For source $V_{2}$, the (64·32,64·10) SPC is constructed via Gaussian approximation where the underlying channel is BI-AWGN channels with equivalent capacity $C_{equ2}=\frac{K}{N}\cdot \left(1-H\left(v_{2}^{j}|v_{1}^{j}\right)\right)+\frac{N-K-P}{N}C_{2}=\frac{K}{2N}+\frac{N-K-P}{N}C_{2}$. Since the conventional puncturing scheme is not applicable in this situation, 64·11 parity bits of source $V_{2}$ are randomly punctured.

### 5.1. Performance under SC Decoders

**Figure 7:**
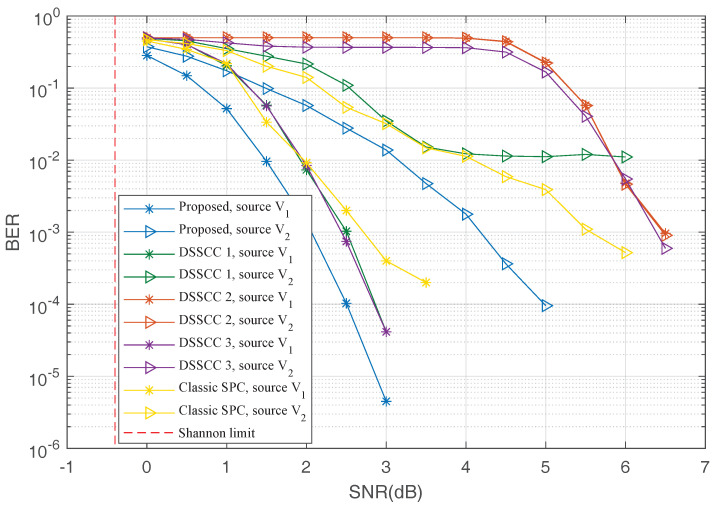
The bit error rate (BER) performance under the SC decoder.

First, we investigate the performance of the proposed scheme, DSSCC schemes, and the DJSCC scheme with classic SPCs under the SC decoder. Figure 7 shows the BER performance versus the SNR of transmission channels. In this figure, lines labeled by “Proposed...” represent BERs for the proposed scheme, lines labeled by “DSSCC...” represent BERs for DSSCC schemes with different values of $R_{si},R_{ci}$ shown in Table 1, and lines labeled by “Classic SPC...” represent the DJSCC scheme with classic SPCs. From these simulation results, we can see that the proposed scheme outperforms DSSCC schemes and the DJSCC scheme with classic SPCs by approximately 0.2∼2 dB. It can be observed that an “error-floor” of “DSSCC 1” starts from 3.5 dB, which is completely caused by the source joint decoder (channel decoders get almost error-free codewords from 3.5 dB to 5 dB). To avoid this error, the higher compressed rate $R_{si}$ is required. Therefore, we do not observe the “error-floor” for “DSSCC 2” and “DSSCC 3”.

### 5.2. Performance under CA-SCL Decoders

For the short or moderate code-length, the performance of polar codes under SC decoder is dissatisfied. To improve the performance, Tal and Vardy proposed CRC-aid SC list (CA-SCL) decoder for polar codes [22]. Next, we investigate the BER performance of the proposed scheme together with two referenced schemes under the CA-SCL decoder. For QSPCs and punctured QSPCs, systematic bits contain 16 bits CRC, i.e., the length of source message is MK−16. At the decoding stage, LLRs of these 16 bits are initialized as 0 and the list size is set as 32. Taking CRC into consideration, the rates of the DJSCC scheme become $\tilde{R}=\left(2.256,1.128\right)$ in channel-use/source-symbol. In DSSCC schemes, information bits of both DSC and CC contain 16 bits CRC and the list size is also set as 32.

**Figure 8:**
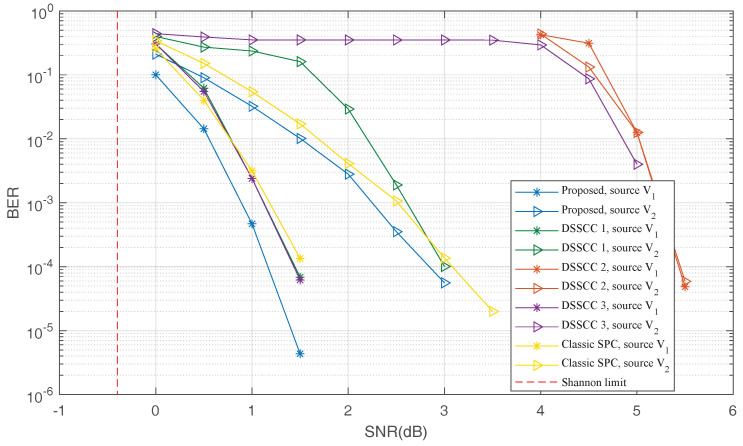
The BER performance under the CRC-aid SC list (CA-SCL) decoder.

Figure 8 shows the BER performance under the CA-SCL decoder. We can see that compared with the SC decoder, the performances of all schemes are improved significantly. Due to the reasonable rate allocation, the performance of “DSSCC 1” is the best and very close the performance of the proposed scheme. However, the performances of other two DSSCC schemes are dissatisfied. It can be observed that the proposed scheme outperforms “DSSCC 1” and the DJSCC scheme with classic SPCs by approximately 0.2∼1 dB.

### 5.3. Complexity

For each distributed source, the encoding complexities of both two referenced schemes are O(NlogN). The encoding complexity of the proposed scheme is slightly larger, which is O(N+NlogN). Due to low encoding complexity, these schemes are well-suited for WSNs.

**Figure 9:**
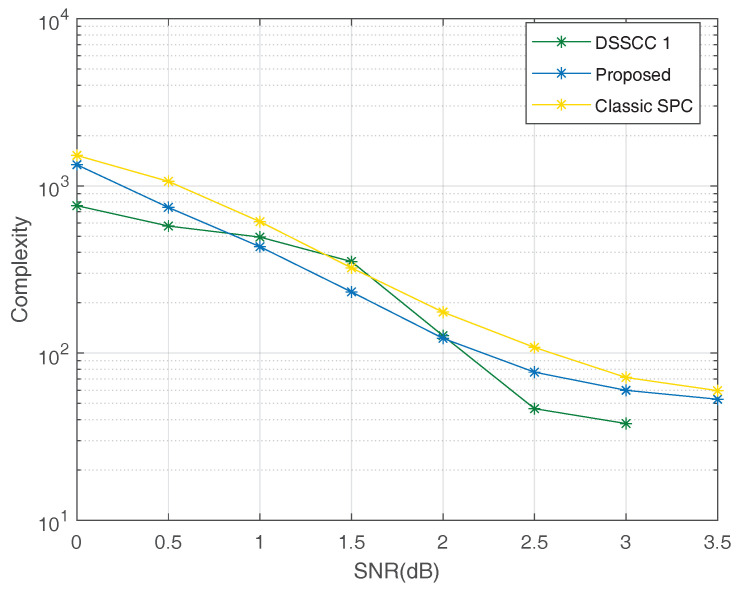
The decoding complexity (per source-symbol) under adaptive CA-SCL decoders.

To investigate decoding complexity, we consider adaptive CA-SCL decoders [23] where the list size is expanded if decoding error is detected by CRC. These kinds of decoders have $O(\bar{L}N\log N)$ complexity, where $\bar{L}$ is average list size. In the simulations, the allowable maximum list size is set as $L_{max}=32$ and the average list size $\bar{L}$ for decoding both two sources is recorded. The complexity (per source-symbol) can be approximated as $O(\frac{1}{MK}\cdot \bar{L}N\log N)$ for the proposed scheme and DJSCC scheme with classic SPCs, and $O(\frac{1}{N_{s}}\cdot \bar{L}N\log N)$ for DSSCC schemes. As shown in Figure 9, for the high SNR region and the low SNR region, the DSSCC1 has lower decoding complexity while the proposed scheme has lower decoding complexity for the middle SNR region. In general, three schemes have similar decoding complexity under adaptive CA-SCL decoders.

## 6. Conclusions

In this paper, we construct two kinds of polar-like codes (QSPCs and punctured QSPCs), which can be used for JSCC with side information. We then proposed a DJSCC scheme based on QSPC and punctured QSPC. In this scheme, we have transformed the optimization transmission problem into a problem of optimizing JSCC with side information at the receiver, which can be solved by QSPCs and punctured QSPCs. For the infinite code-length, we have proved that the proposed scheme is asymptotically optimal. For the finite code-length, the simulation results have verified that the proposed scheme outperforms the DSSCC scheme with polar codes and the DJSCC scheme with classic SPCs.

## Figures and Tables

**Table 1:** Distributed separate source-channel coding (DSSCC) schemes with different $R_{si},R_{ci}$.

	$R_{s1}$	$R_{s2}$	$R_{c1}$	$R_{c2}$	$\tilde{R}$
DSSCC 1	1024/1024	650/1024	465/1024	591/1024	(2.2, 1.1)
DSSCC 2	900/1024	900/1024	409/1024	818/1024	(2.2, 1.1)
DSSCC 3	1024/1024	900/1024	465/1024	818/1024	(2.2, 1.1)
